# Adiponectin and AMP kinase activator stimulate proliferation, differentiation, and mineralization of osteoblastic MC3T3-E1 cells

**DOI:** 10.1186/1471-2121-8-51

**Published:** 2007-11-29

**Authors:** Ippei Kanazawa, Toru Yamaguchi, Shozo Yano, Mika Yamauchi, Masahiro Yamamoto, Toshitsugu Sugimoto

**Affiliations:** 1 Department of Internal Medicine 1, Shimane University School of Medicine, Japan

## Abstract

**Background:**

Adiponectin is a key mediator of the metabolic syndrome that is caused by visceral fat accumulation. Adiponectin and its receptors are known to be expressed in osteoblasts, but their actions with regard to bone metabolism are still unclear. In this study, we investigated the effects of adiponectin on the proliferation, differentiation, and mineralization of osteoblastic MC3T3-E1 cells.

**Results:**

Adiponectin receptor type 1 (AdipoR1) mRNA was detected in the cells by RT-PCR. The adenosine monophosphate-activated protein kinase (AMP kinase) was phosphorylated by both adiponectin and a pharmacological AMP kinase activator, 5-amino-imidazole-4-carboxamide-riboside (AICAR), in the cells. AdipoR1 small interfering RNA (siRNA) transfection potently knocked down the receptor mRNA, and the effect of this knockdown persisted for as long as 10 days after the transfection. The transfected cells showed decreased expressions of type I collagen and osteocalcin mRNA, as determined by real-time PCR, and reduced ALP activity and mineralization, as determined by von Kossa and Alizarin red stainings. In contrast, AMP kinase activation by AICAR (0.01–0.5 mM) in wild-type MC3T3-E1 cells augmented their proliferation, differentiation, and mineralization. BrdU assay showed that the addition of adiponectin (0.01–1.0 μg/ml) also promoted their proliferation. Osterix, but not Runx-2, appeared to be involved in these processes because AdipoR1 siRNA transfection and AICAR treatments suppressed and enhanced osterix mRNA expression, respectively.

**Conclusion:**

Taken together, this study suggests that adiponectin stimulates the proliferation, differentiation, and mineralization of osteoblasts via the AdipoR1 and AMP kinase signaling pathways in autocrine and/or paracrine fashions.

## Background

Cumulative evidence has shown that there is a positive correlation between bone mineral density (BMD) and fat mass, suggesting that body fat and bone mass are related to each other [1-6]. Several studies on adipocyte function have revealed that not only is adipose tissue an energy-storing organ but it also secretes a variety of biologically active molecules, which are named adipocytokines [7]. Adiponectin is one of the adipocytokines specifically and highly expressed in adipose tissue. It is also abundantly present in plasma [8] and has been proposed to play important roles in the regulation of energy homeostasis and insulin sensitivity [9-11]. Two adiponectin receptor subtypes, adiponectin receptor type 1 and 2 (AdipoR1 and AdipoR2), have been identified; these mediate the biological effects of adiponectin [12]. AdipoR1 is abundantly expressed in the muscle, whereas AdipoR2 is predominantly expressed in the liver [12]. Several studies indicate that AdipoR1 and AdipoR2 serve as receptors for globular and full-length adiponectin, respectively, and that their stimulation by adiponectin results in increased AMP-activated protein kinase (AMP kinase) and PPARα ligand activities as well as fatty acid oxidation and glucose uptake in the liver and skeletal muscle [12-14]. Moreover, the expression levels of AdipoR seem to be very important for adiponectin sensitivity. Obesity decreases AdipoR expression levels, thereby reducing adiponectin sensitivity and enhancing insulin resistance [15]. Genetic variations in AdipoR have been reported to be linked to a common genetic predisposition to insulin resistance or type 2 diabetes mellitus [16].

Recently, it has been demonstrated that adiponectin and its receptors are also expressed in osteoblasts [17-20], suggesting that adiponectin may carry signals and may have a function in bone tissue as well. However, there have been only few studies on the physiological actions of adiponectin on osteoblasts, and thus this issue requires further clarification.

The present study was undertaken to investigate the abovementioned issue in osteoblastic MC3T3-E1 cells using AdipoR inhibition by its small interfering RNA (siRNA) and AMP kinase activation by its pharmacological activator 5-aminoimidazole-4-carboxamide-1-β-D-ribonucleoside (AICAR) [21,22]. The results showed that adiponectin promoted the proliferation, differentiation, and mineralization of MC3T3-E1 cells possibly via the AdipoR1 and AMP kinase pathways, suggesting that adiponectin might cause osteoblastogenesis via AMP kinase activation.

## Results

### Expression of adiponectin receptors and knockdown by siRNA transfection

**Figure 1:**
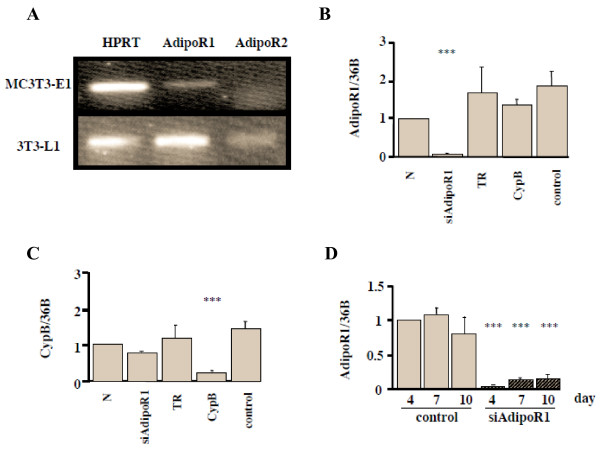
Adiponectin R1 and R2 expression in MC3T3-E1 cells and effects of siRNA-AdipoR1 transfection. (A) Adiponectin receptor expression in MC3T3-E1 cells. Total RNA from the cells was subjected to RT-PCR. HPRT, house keeping gene, and AdipoR1 were visualized in a 2% agarose gel stained with ethidium bromide. AdipoR1 mRNA but not AdipoR2 was expressed in MC3T3-E1 cells, while both of them were expressed in 3T3-L1 cells, which were examined as a positive control. (B) Confirmation of the effect of siRNA-AdipoR1. The siRNA restrained only siAdipoR1, showing that its knock down effect was specific (p < 0.001). N; 0.2% BSA, TR; transfection reagent, CypB; transfection of siRNA-Cyclophilin B (CypB), control; transfection of non-targeting siRNA. (C) Confirmation of the effect of siRNA-CypB. (D) The durability of siRNA-AdipoR1. Total RNA was collected at 4, 7, 10 days after siRNA transfection. The knock down effect of siRNA-AdipoR1 was sustained as long as 10 days after transfection at the expression level of 14.9% of the control. Results are expressed as the mean ± SEM fold increase (n = 5) over control values. * p < 0.05, ** p < 0.01, *** p < 0.001.

We first investigated AdipoR1 and AdipoR2 expressions in MC3T3-E1 cells. By using RT-PCR, we confirmed that AdipoR1 mRNA, but not AdipoR2, was expressed in MC3T3-E1 cells (Fig. 1A). To elucidate the action of adiponectin through AdipoR1, we performed experiments using an RNA interference to block the receptor expression. Real-time PCR revealed that a treatment with 50-nM siRNA-AdipoR1 blocked the expression of AdipoR1 mRNA (Fig. 1B), while a treatment with cyclophilin B (CypB) siRNA blocked the expression of CypB mRNA (Fig. 1C), showing that the knockdown effect of siRNA-AdipoR1 was specific. The effect of knockdown by siRNA-AdipoR1 persisted for as long as 10 days after the transfection, when the expression level was still suppressed by 85.1% in comparison to that in the control (Fig. 1D).

### Effect of AdipoR1 knockdown on the differentiation and mineralization of MC3T3-E1 cells

**Figure 2:**
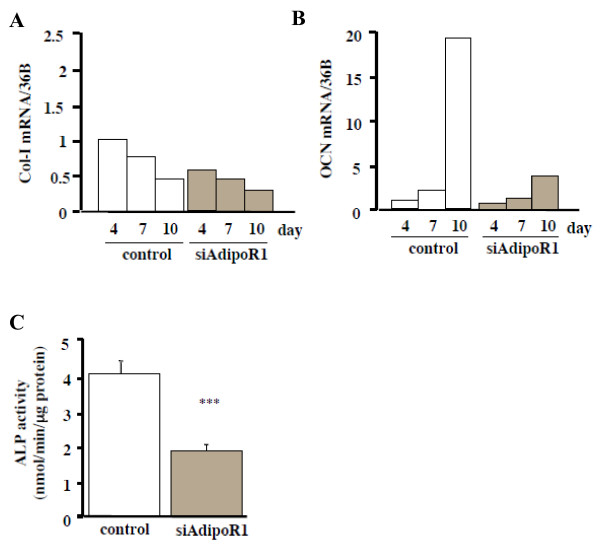
Effects of siRNA-AdipoR1 transfection on the differentiation of MC3T3-E1 cells. Total RNA was collected at 4, 7, 10 days after siRNA-AdipoR1 transfection. Col-I (A) and OCN (B) mRNA expression were decreased by blocking the receptor expression. Results are expressed as fold increase over the control values at 4 days. The result was the representative of five different experiments. (C) ALP activity of siRNA-AdipoR1-transfected MC3T3-E1 cells. ALP activity was evaluated biochemically. ALP activity was significantly decreased compared to the control (*** p < 0.001).

**Figure 3:**
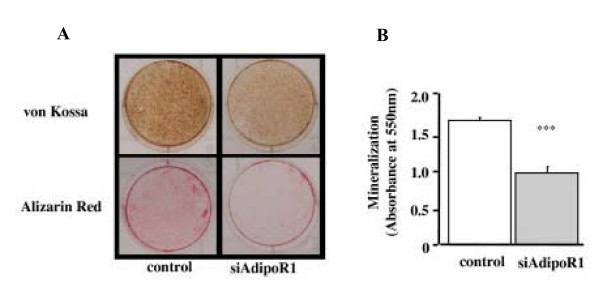
Effects of siRNA-AdipoR1 transfection on the mineralization of MC3T3-E1 cells. Mineralization of MC3T3-E1 was evaluated by von Kossa and Alizarin red stainings. (A) Plate view of von Kossa and Alizarin red stainings in cultured MC3T3-E1. (B) Quantification of Alizarin red staining via extraction with etylpyridinium chloride. The amount of released dye was quantified by microplate reader at 550 nm. Mineralizarion was significantly decreased in siRNA-AdipoR1 treatment compared to the control (*** p < 0.001). The result was the representative of three different experiments.

To examine the effect of AdipoR1 knockdown on MC3T3-E1 cells, we analyzed the differentiation and mineralization of the siRNA-transfected cells. The total RNA was collected 4, 7, and 10 days after the siRNA treatment. Real-time PCR showed reduced collagen-I (Col-I) and osteocalcin (OCN) mRNA levels by the siRNA-AdipoR1 treatments (Figs. 2A and 2B, respectively). After the siRNA-AdipoR1 transfection, the ALP activity was significantly lower than that of the control on day 14 (p < 0.001) (Fig. 2C), and both von Kossa and Alizarin red stainings showed that the mineralization was apparently inhibited by siRNA-AdipoR1 on day 28 (Fig. 3A). The quantification of the Alizarin red staining showed that this inhibition was significant (p < 0.001) (Fig. 3B). These results suggested that adiponectin promotes the differentiation and mineralization of MC3T3-E1 cells via AdipoR1.

### Effects of adiponectin and AICAR on the AMP kinase signaling pathway in MC3T3-E1 cells

**Figure 4:**
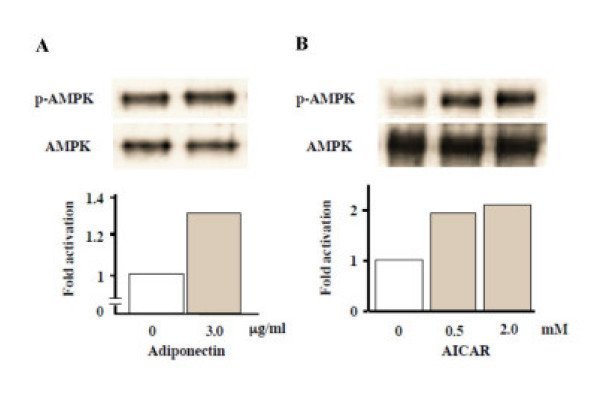
Effects of recombiant adiponectin and AICAR treatments on AMP kinase activation in MC3T3-E1 cells. MC3T3-E1 cells were starved in serum-free medium for 2 h and then stimulated by addition of serum-free medium with 3.0 μg/ml recombinant adiponectin or 0.5–2.0 mM AICAR. Whole cell lysates were collected after 5 min incubation for Western blotting. Immunoprecipitation and immunoblotting were performed as described in MATERIALS AND METHODS. The lower panels show the intensity of phosphorylated AMP kinase (p-AMPK) that was measured using the NIH Image Program and was normalized using the total AMP kinase (AMPK) signal. Recombinant adiponectin (A) and AICAR (B) activated AMP kinase rapidly. The result was the representative of three different experiments.

To determine whether or not adiponectin or AICAR, a pharmacological AMP kinase activator, could phosphorylate AMP kinase, MC3T3-E1 cells were incubated with 3 μg/ml recombinant adiponectin or 0.5–2.0 mM AICAR. A western blot analysis revealed that both adiponectin and AICAR were able to phosphorylate AMP kinase (Figs. 4A and 4B, respectively).

### Effect of AMP kinase activation on the differentiation and mineralization of MC3T3-E1 cells

**Figure 5:**
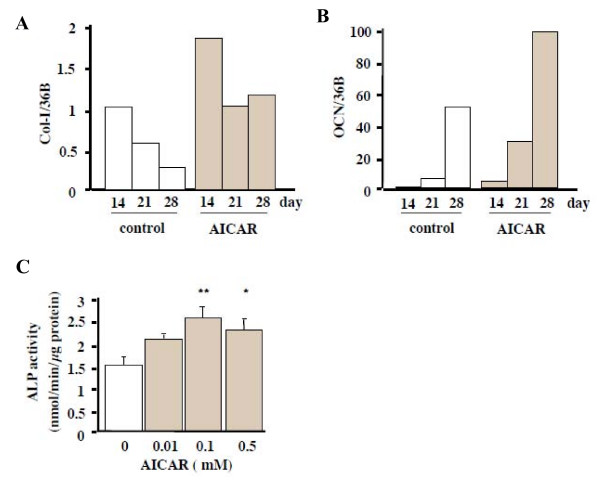
Effects of an AICAR treatment on the differentiation of MC3T3-E1 cells. AICAR (0.5 mM), a AMP kinase stimulator, was added after the cells reached confluency. Total RNA was collected at 14, 21, 28 days. Col-I (A) and OCN (B) mRNA expression were increased by the AICAR treatment. Results were expressed as fold increase over the control values at 14 days. The result was the representative of three different experiments. (C) ALP activity of MC3T3-E1 cells by an AICAR treatment. ALP activity was evaluated biochemically. ALP activity was increased compared to the control significantly (* p < 0.05, and ** p < 0.01).

**Figure 6:**
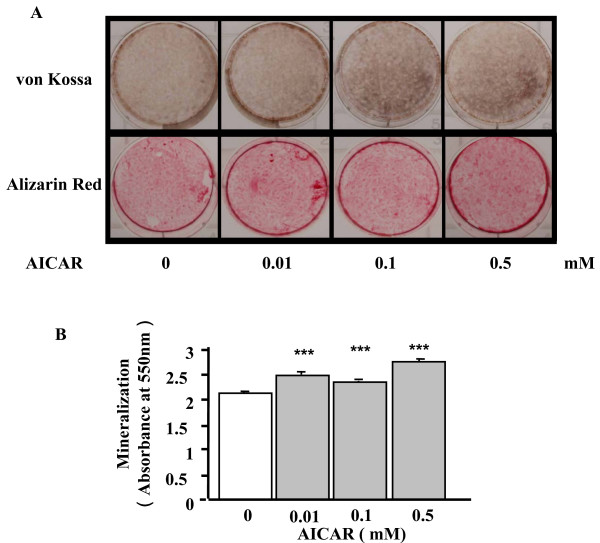
Effects of an AICAR treatment on the mineralization of MC3T3-E1 cells. Mineralization of MC3T3-E1 was evaluated by von Kossa and Alizarin red stainings. (A) Plate view of von Kossa and Alizarin red stainings in cultured MC3T3-E1 cells. (B) Quantification of Alizarin red staining via extraction with etylpyridinium chloride. The amount of released dye was quantified by microplate reader at 550 nm. Mineralization was significantly increased in the AICAR treatment compared to the control (*** p < 0.001). The result was the representative of three different experiments.

Because the results above demonstrated that adiponectin and AICAR activated the AMP kinase signaling pathway in wild-type MC3T3-E1 cells, we examined whether or not the activation of this pathway would lead to the differentiation and mineralization of the cells. AICAR (0.5 mM) was added after the cells reached confluency, and the total RNA was collected on days 14, 21, and 28. Real-time PCR showed that in contrast to the results for siRNA-AdipoR1, the AICAR treatments increased the expression levels of Col-I and OCN mRNA (Figs. 5A and 5B, respectively). ALP activities were significantly greater than those of the control at 0.1 and 0.5 mM concentrations of AICAR and in a 14-day culture (p < 0.05) (Fig. 5C). Moreover, both von Kossa and Alizarin red stainings showed that the mineralization of MC3T3-E1 cells was augmented by 0.01–0.5 mM AICAR on day 28 (Fig. 6A). The quantification of the Alizarin red staining showed that these augmentations were significant (p < 0.001) (Fig. 6B). Thus, the activation of AMP kinase seemed to promote the differentiation and mineralization of MC3T3-E1 cells.

### Effect of AdipoR1 knockdown and AICAR treatments on Runx-2 expression in MC3T3-E1 cells

**Figure 7:**
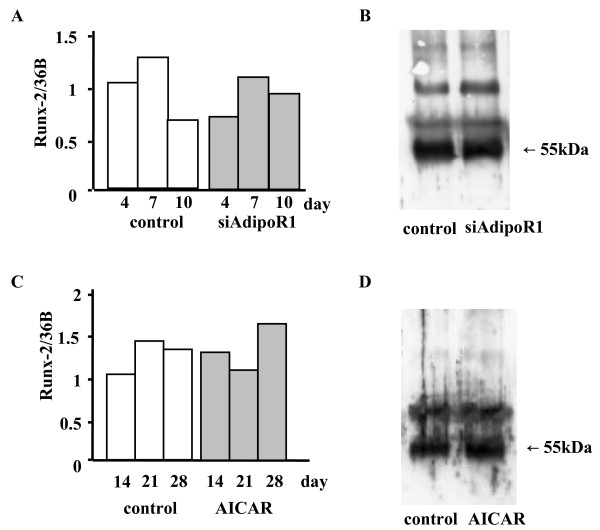
Effects of siRNA-AdipoR1 transfection or an AICAR treatment on Runx-2 mRNA and protein expressions in MC3T3-E1 cells. (A and B) Total RNA was collected at 4, 7, 10 days after siRNA-AdipoR1 transfection, and cell lysate was collected at 7 days after transfection. Real-time PCR and immunoprecipitation were performed as described in MATERIALS AND METHODS. Neither Runx-2 mRNA (A) nor its protein (B) expressions were changed by blocking the receptor expression. (C and D) AICAR (0.5 mM), an AMP kinase stimulator, was added after the cells reached confluency. Total RNA was collected at 14, 21, 28 days, and cell lysate was collected at 14 days. Neither Runx-2 mRNA (C) nor its protein (D) expressions were changed by AICAR. The result was the representative of five different experiments.

Since Runx-2 (also called cbfa1, PEBP2α A) is an important transcription factor that is involved in osteoblastic differentiation and mineralization [23], we investigated whether or not adiponectin and AICAR modulate its expression. There were little or no changes in the Runx-2 mRNA levels, as determined by real-time PCR, or its protein levels (55-kDa band), as determined by immunoblotting under either siRNA-AdipoR1 transfection (Figs. 7A and 7B, respectively) or AICAR treatment (Figs. 7C and 7D, respectively).

### Effect of AdipoR1 knockdown and AICAR treatments on osterix expression in MC3T3-E1 cells

**Figure 8:**
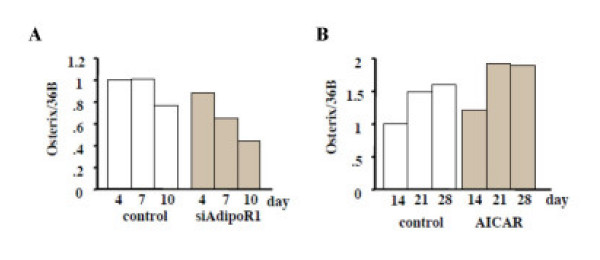
Effects of siRNA-AdipoR1 transfection or AICAR treatments on Osterix mRNA expression in MC3T3-E1 cells. Total RNA was collected at 4, 7, 10 days after siRNA-AdipoR1 transfection. Real-time PCR was performed as described in MATERIALS AND METHODS. Osterix mRNA expressions was suppressed by blocking the receptor expression (A). AICAR (0.5 mM), an AMP kinase stimulator, was added after the cells reached confluency. Total RNA was collected at 14, 21, 28 days. Osterix mRNA expressions was enhanced by AICAR (B). The result was the representative of five different experiments.

Since osterix (Osx) is also an important transcription factor that is involved in osteoblastic differentiation and mineralization, we investigated whether or not adiponectin and AICAR modulate its expression. Real-time PCR showed that Osx mRNA expressions were suppressed by siRNA-AdipoR1 transfection (Figs. 8A) and enhanced by AICAR treatments (Figs. 8B).

### Effects of adiponectin and AICAR on the proliferation of MC3T3-E1 cells

**Figure 9:**
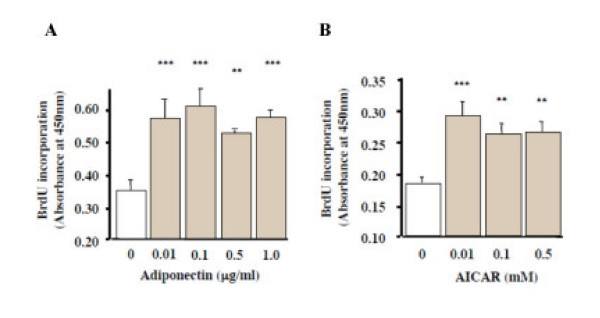
Effects of recombinant adiponectin and AICAR treatments on BrdU incorporation by MC3T3-E1 cells. Cells were exposed to 0.01–1.0 μg/ml adiponectin or 0.01–0.5 mM AICAR for 24 h. Cell proliferation was determined by measuring the amount of incorporated BrdU. Proliferation was significantly promoted by both low dose adiponectin (A) and AICAR (B) treatments (p < 0.01). The results were the representative of three different experiments.

We found that after 24-h incubation with adiponectin or AICAR, BrdU incorporation was significantly enhanced in MC3T3-E1 cells (p < 0.01) (Figs. 9A and 9B, respectively). These results showed that adiponectin and the AMP kinase activator stimulated the proliferation of the cells.

## Discussion

Recent studies have shown that adiponectin and its receptors were detected not only in adipocytes but also in bone-forming cells [17-20]. However, little is known about the physiological action of adiponectin on osteoblasts. In this study, we demonstrated that osteoblastic MC3T3-E1 cells expressed AdipoR1, but not AdipoR2. We found that the suppression of AdipoR1 expression by its siRNA inhibited the differentiation and mineralization of the cells, and we suggested that adiponectin promoted these processes through the action of AdipoR1 on the osteoblasts. We also found that adiponectin activated AMP kinase in the cells, and that AICAR, a pharmacological AMP kinase activator [21,22], promoted the differentiation and mineralization of the cells. Both adiponectin and AICAR also stimulated the proliferation of the cells. Taken together, adiponectin seemed to promote the proliferation, differentiation, and mineralization of osteoblastic MC3T3-E1 cells possibly via the AdipoR1 and AMP kinase signaling pathway. Recent studies have also shown that adiponectin promotes osteoblast proliferation [17,19] and exerts an enhancing action on human osteoblast differentiation and mineralization [19]. Although we did not carry out experiments using the inhibitor or siRNA of AMP kinase, our results seem to be consistent with those reported in these studies. Further, we disclosed the involvement of the AMP kinase signaling pathway in the stimulatory actions of adiponectin on osteoblasts.

Luo et al. have reported that the adiponectin concentration in fetal bovine serum (FBS) was approximately 60 μg/ml, which was about six-fold higher than that in human serum. They have shown that the addition of FBS into the medium actually enhances the ALP activity of primary human osteoblasts; however, the addition of adiponectin-free FBS does not enhance the activity [19]. Therefore, the influence of adiponectin, which is present in high concentrations in FBS, must be ignored when the differentiation and mineralization of osteoblasts in long cultures are examined. In this study, we knocked down the expression of AdipoR1 by transfecting its siRNA and investigated how the lack of adiponectin action in the microenvironment of MC3T3-E1 cells would affect the cell functions. There seem to be two advantages of using MC3T3-E1 cells as a model system to study the role of adiponectin signaling on osteoblast growth and differentiation. First, since the cells produce adiponectin endogenously [17], there is no need to add adiponectin to the cultures. Second, since the present study and a previous one [17] showed that the cells do not express AdipoR2, data interpretation will not be complicated by the compensatory effect of AdipoR2 after the siRNA knockdown of AdipoR1. By reducing AdipoR1 expression through its siRNA transfection, we were able to demonstrate that endogenously produced adiponectin was important in osteoblast proliferation and differentiation as well as in matrix mineralization.

Shinoda et al. have shown that there were no abnormalities in the bone mass or turnover of either adiponectin-deficient mice or adiponectin-transgenic mice that overexpressed adiponectin in the liver [20]. They have reported that circulating adiponectin suppresses osteoblastogenesis through the endocrine pathway, while local adiponectin stimulates it through the autocrine/paracrine pathway [20]. Additionally, they have suggested that these contradictory effects of adiponectin might explain no apparent bone changes in its gene-modified mice. In this study, we found that the blockage of the AdipoR1 expression by transfecting its siRNA inhibited the differentiation and mineralization of MC3T3-E1 cells. Since this siRNA transfection obstructed not only the autocrine/paracrine pathway of adiponectin but also its endocrine pathway, cytokine might play an important role in the activation of osteoblasts in the microenvironment of bone marrow.

Yokota et al. have shown that adiponectin is also found in bone marrow fat cells [24], and Berner et al. have shown that the level of adiponectin is higher in diluted bone marrow fluid than in the serum of rats [17]. These facts suggest that fat cells as well as bone cells might contribute to the production and high concentration of adiponectin found in bone marrow. Because adiponectin receptors are known to be expressed ubiquitously [12], it is possible that adiponectin can influence not only osteoblasts but also other lineages of bone marrow cells. In fact, adiponectin has been shown to exert an inhibitory effect on B lymphopoiesis and a stimulatory effect on myelopoiesis in bone marrow [24,25]. Thus, further studies seem to be necessary to clarify the interplay among osteoblasts and other cells in the biological actions of adiponectin in the microenvironment of bone marrow.

Runx-2 has been identified as an important transcription factor that is involved in osteoblast differentiation and mineralization [23]. Hence, it is possible that the stimulatory actions of adiponectin and AICAR on MC3T3-E1 cells in this study might be mediated by this transcription factor. However, neither of these agents affected mRNA or protein expressions of Runx-2, suggesting that the transcription factor might be not involved in the stimulatory effects of adiponectin on MC3T3-E1 cells. On the other hand, there remains a possibility that the Runx-2 activity can be altered by the adiponectin signaling pathway. It has been demonstrated that Runx-2 was activated by Erk via phosphorylation [26,27]. Although the Erk activity was reported to be not altered by adiponectin [19], it is not clear from our current experiments whether the adiponectin downstream effector AMP kinase can phosphorylate Runx-2 either directly or indirectly. Alternatively, we indicated that Osx mRNA expression was enhanced by adiponectin and AICAR. Although Osx is generally regarded as the downstream effector of Runx-2, the upregulation of Osx mRNA has been reported without the alteration of Runx-2 mRNA levels or in the absence of Runx-2 [28,29], suggesting that Osx and Runx-2 can be differently regulated and Osx can modulate osteoblastic function independent of Runx-2. Moreover, Shinoda et al. have recently shown that adiponectin activates the intracellular signaling of insulin in bone marrow cells through the phosphorylation of IRS-1 and Akt, the main downstream molecules of insulin [20], thereby suggesting that insulin/IGF-I signaling pathways might be alternative candidates for mediating the stimulatory effects of adiponectin on osteoblasts.

The biological effects of adiponectin are known to be mediated through the two adiponectin receptor subtypes AdipoR1 and R2 [12], which subsequently activate AMP kinase, PPARα ligand, and mitogen-activated protein kinase (MAPK), and regulate fatty-acid oxidation and glucose uptake [12]. AMP kinase is a highly conserved heterotrimeric signaling kinase responsive to hypoxia, exercise, and cellular stress, and it is associated in a variety of cellular responses, including suppression of gluconeogenesis in the liver, promotion of glucose uptake in skeletal muscle, inhibition of fatty acid and sterol synthesis, increase in fatty acid oxidation, and inhibition of lipolysis [30-34]. In contrast, there exists little or no information about the potential roles of this signaling molecule in bone metabolism. In this study, we found that AMP kinase activation promoted the proliferation, differentiation, and mineralization of MC3T3-E1 cells. AMP kinase is known to have the ability to phosphorylate and inactivate 3-hydroxy-3-methylglutaryl-CoA (HMG-CoA) reductase, a key regulatory enzyme in the synthesis of cholesterol and other isoprenoid compounds [35]. Statins, which also inactivate HMG-CoA reductase, have been shown to promote osteoblastogenesis via cellular BMP-2 production [36,37]. Thus, further studies are necessary to investigate whether or not the activation of AMP kinase would inactivate HMG-CoA reductase in MC3T3-E1 cells and enhance their differentiation and mineralization.

A couple of studies have revealed that there are interplays between the AMP kinase pathway and the MAPK pathway in the cellular responses of osteoblasts as well as the islet cells in the pancreas. Kefas et al. have previously shown that the prolonged activation of AMP kinase by AICAR promotes apoptosis of the beta cells in the islets through a sequential activation of JNK [38,39]. Luo et al. have reported that the adiponectin-induced proliferative response of human osteoblasts was mediated by the AdipoR/JNK pathway, while the differentiation response was mediated via the AdipoR/p38 MAPK pathway [19]. Although we found that the stimulatory effects of adiponectin on MC3T3-E1 cells might be mediated by AMP kinase, these previous results suggest that the involvement of MAPK or other pathways must also be investigated in future.

## Conclusion

Our results suggest that adiponectin stimulates the proliferation, differentiation, and mineralization of osteoblastic MC3T3-E1 cells via the AdipoR1 and AMP kinase signaling pathways in autocrine and/or paracrine fashions in the microenvironment of cultured cells. Our findings seem to be in accord with previous ones that showed that adiponectin had a stimulatory action on osteoblasts [17-20] and they seem to extend this notion by showing the involvement of the AMP kinase pathway in these processes.

## Methods

### Materials

Cell culture medium and supplements were purchased from Gibco-BRL (Rockville, MD). Recombinant mouse globular adiponectin, which is produced in E. coli, and AICAR were purchased from Peprotech EC (London, UK) and Sigma-Aldrich (St. Louis, MO), respectively. For Western blot analysis, mouse anti-phosphorylated-AMP kinase and mouse anti-AMP kinase antibodies were purchased from Cell Signaling Technology (Beverly, MA), and anti-Runx-2 antibody was purchased from Santa Cruz Biotechnology (Santa Cruz, CA). A secondary antibody (rabbit anti-mouse IgG) for immunoblotting was obtained from Sigma (St. Louis, MO). The enhanced chemiluminescence (ECL) system for Western blot analysis was purchased from Amersham (Arlington Heights, IL). All other chemicals were of the highest grade available commercially.

### Cell cultures

MC3T3-E1 cells, a clonal osteoblastic cell line isolated from calvariae of late stage mouse embryo [40], were kindly provided by Kobe University Graduate School of Medicine, Japan. 3T3-L1 cells, a clonal preadipocyte cell line, were purchased from RIKEN CELL BANK (Ibaragi, Japan). MC3T3-E1 cells were cultured in α-minimum essential medium (α-MEM). This medium was supplemented with 10% FBS and 1% penicillin-streptomycin (GIBCO-BRL) in 5% CO_2 _at 37°C. The medium was changed twice a week. For mineralization assay, MC3T3-E1 cells were cultured in α-MEM containing 10% FBS, 1% penicillin-streptomycin, and 10 mM β-glycerophosphate for 3 weeks after reaching confluence.

### RNA interference for adiponectin receptor

RNA interference was used to down-regulate the expression of AdipoR1 in MC3T3-E1 cells. SMARTpool small interfering RNA (siRNA) and SMARTpool reagents for AdipoR1, CypB and nonspecific control siRNA duplexes were designed and synthesized by Customer SMARTpool siRNA Design from Dharmacon (Lafayette, CO). For gene knock down experiments, MC3T3-E1 cells were plated in 6 cm dish and cultured for 48 hr in α-MEM containing 10% FBS and antibiotics. Next, after 24 hr incubation in medium without antibiotics, cells were transfected with siRNAs (50 nM) using transfection reagent according to the manufacture's instructions. After another 48 hr of culture, cells were recultured in another in α-MEM containing 10% FBS and antibiotics.

### RT-PCR analysis of osteoblastic MC3T3-E1 cells mRNA for identification of adiponectin receptors

For investigating the expression of AdipoR mRNA in osteoblastic MC3T3-E1 cells, reverse transcription (RT)-polymerase chain reaction (PCR) was performed. Total RNA was taken from cultured MC3T3-E1 cells using Trizol reagent (Invitrogen, San Diego, CA) according to the manufacturer's recommended protocol. Two μg total RNA was employed for the synthesis of single-stranded cDNA (cDNA synthesis kit; Invitrogen). For amplification of AdipoR1 and R2, primers were selected as previously reported (Table 1) [41]. The PCR condition was 95°C for 15 s, 60°C for 30 s, and 72°C for 1 min for 34 cycles. As an internal standard, cDNA for the housekeeping gene hypoxanthine phosphoribosyltransferase (HPRT) was amplified using specific primers. PCR products were separated by electrophoresis on 2% agarose gel and visualized by ethidium bromide staining with ultraviolet (UV) light using Electronic UV trans-illuminator (Toyobo CO. Ltd, Tokyo, Japan).

**Table 1:** Gene names and its primers.

Gene name	Primers	Accession no.
mAdipoR1	CTTCTACTGCTCCCCACAGC	NM_028320
	TCCCAGGAACACTCCTGCTC	
mAdipoR2	CCACACAACACAAGAATCCG	XM_132831
	CCCTTCTTCTTGGGAGAATGG	
mCypB	TCGGAGCGCAATATGAAGGT	BC013061
	TTCTTCTTATCGTTGGCCACG	
mCol-I	AACCCGAGGTATGCTTGATCT	NM_007742
	CCAGTTCTTCATTGCATTGC	
mOCN	TGCTTGTGACGAGCTATCAG	L24431
	GAGGACAGGGAGGATCAAGT	
mRunx-2	AAGTGCGGTGCAAACTTTCT	NM_009820
	TCTCGGTGGCTGGTAGTGA	
mOsterix	CCCTTCTCAAGCACCAATGG	AF184902
	AGGGTGGGTAGTCATTTGCATAG	
m36B4	AAGCGCGTCCTGGCATTGTCT	NM_007475
	CCGCAGGGGCAGCAGTGGT	
HPRT	CAGTCCCAGCGTCGTGATTA	J00423
	AGCAAGTCTTTCAGTCCTGTC	

Primer pairs used for studying various genes.

m; mouse, AdipoR; adiponectin receptor, CypB; cyclophilin B, Col-I; collagen type I, OCN; osteocalcin, HPRT; hypoxanthine phosphoribosyltransferase.

36B4 and HPRT are housekeeping genes.

### Real-time PCR quantification of gene expression

SYBR green chemistry was used to perform quantitative determination for the mRNAs for AdipoR1, collagen I, osteocalcin, Runx-2, and a house keeping gene, 36B4, following an optimized protocol [42,43]. The design of sense and antisense oligonucleotide primers was based on published cDNA sequences using the Primer Express (version 2.0.0, Applied Biosystems). Primer sequences were listed in Table 1, and cDNA was synthesized with the reverse transcription kit (Invitrogen). Real-time PCR was performed using 1 μl of cDNA in a 25 μl reaction volume with ABI PRISM 7000 (PE Applied Biosystems Inc.). The double-stranded DNA-specific dye SYBR Green I was incorporated into the PCR buffer provided in the QuantiTech SYBR PCR kit (QIAGEN, Valencia, CA) to allow for quantitative detection of the PCR product. The temperature profile of the reaction was 95°C for 15 min, 40 cycles of denaturation at 94°C for 15 sec, and annealing and extension at 60°C for 1 min. 36B4 was used to normalize differences in RNA isolation [44], RNA degradation, and the efficiencies of the reverse transcription.

### Assay of alkaline phosphatase activity

After reaching confluence, cells in 24-well plates were rinsed three times with PBS, and 600 μl of distilled water were added to each well and sonicated. The protein assay was performed with the bicinchoninic acid (BCA) protein assay reagent (Pierce, Rockford, IL). Alkaline phosphatase (ALP) activity was assayed by a method modified from that of Lowry et al. [45]. In brief, the assay mixtures contained 0.1 M 2-amino-2-methyl-1-propanol, 1 mM MgCl_2_, 8 mM p-nitrophenyl phosphate disodium, and cell homogenates. After a 4 min of incubation at 37°C, the reaction was stopped with 0.1 N NaOH, and the absorbance was read at 405 nm. A standard curve was prepared with p-nitrophenol. Each value was normalized to the protein concentration.

### Assay of mineralization

Mineralization of MC3T3-E1 cells was determined in 6-well or 12-well plates using von Kossa staining or Alizarin red stainings. The cells were fixed with 95% ethanol and stained with AgNO_3 _by the von Kossa method to detect phosphate deposits in bone nodules [46]. At the same time, the order plates were fixed with ice-cold 70% ethanol and stained with Alizarin red to detect calcification. For quantification, cells stained with Alizarin red (n ≥ 6) were destained with ethylpyridium chloride, then the extracted stain was transferred to a 96-well plate, and the absorbance at 550 nm was measured using a microplate reader, as previously described [47].

### Immunoprecipitation and Immunoblotting

For immunoprecipitation and immunoblotting, MC3T3-E1 cells were plated in 6 cm dish and cultured as described above. After 2 hr of serum starvation, cells were then stimulated with 3 μg/ml of recombinant mouse adiponectin, 0.5–2 mM AICAR, or vehicle for 5 min. Cells were rinsed with ice-cold PBS and scraped on ice into lysis buffer (Cell Signaling Technology) that contained 20 mM Tris-HCl (pH 7.5), 50 mM NaCl, 1 mM EGTA, 1 mM Na_2_EDTA, 1% Triton, 2.5 mM sodium pyrophosphate, 1 mM β-glycerophosphate, 1 mM Na_3_VO_4_, and 1 μg/ml leupeptin. The cell lysates were then sonicated for 30 sec. Nuclei and cell debris were removed by centrifugation (12,000 × g for 10 min), and the resultant total cellular lysate in the supernatant was used for immunoprecipitation. A part of the cell lysates was immunoprecipitated with an anti-AMP kinase antibody or anti-Runx-2 antibody conjugated to protein G-Sepharose (Sigma-Aldrich) overnight at 4°C. The cell lysates with the immunoprecipitation that contained an equivalent amount of protein were electrophoresed by 10% SDS-PAGE and transferred to nitrocellulose membrane (BIO-RAD, Hercules, CA). Immunoblot analysis was performed, as previously described [42]. The blots were incubated overnight at 4°C with gentle shaking with a phospho-AMP kinase antibody or Runx-2 antibody at a 1:200 dilution. The blots were extensively washed with PBS containing 1% Tween 20 (BIO-RAD) and 0.15% dry milk (washing solution) at room temperature and were further incubated with a 1:2000 dilution of horseradish peroxidase-coupled rabbit antimouse IgG in PBS containing 1% Tween 20 for 1 hr at room temperature. The blots were then washed, and the signal was visualized by chemiluminescence according to the manufacturer's protocol. After stripping of phospho-AMP kinase blots, total AMP kinase immunoreactivity was determined in the same membrane. National Institutes of Health (NIH) image software (version 1.62) was used to quantify the signal intensity.

### Proliferation assay

MC3T3-E1 cells (approximately 1,000 cells/well) were seeded out in 96-well plates and cultured for 24 hr. Then the cells were washed twice with serum-free medium before addition of new serum-free medium with or without recombinant mouse adiponectin in the range of 0.01–1.0 μg/ml, and AICAR in the range of 0.01–0.5 mM. After 2 hr, BrdU (5-bromo-2'-deoxyuridine)-labeling solution (Amersham) was added and the cells were cultured for an additional 24 hr. At the end of the pulse, the cells were washed, fixed, and denatured, and an ELISA utilizing an anti-BrdU peroxidase-conjugated antibody was used to measure the amount of incorporated BrdU.

### Statistics

Results are expressed as a mean ± SEM. Statistical evaluations for differences between groups were carried out using one-way analysis of variance (ANOVA) followed by Fisher's protected least significant difference (PLSD). For all statistical tests, a value of P < 0.05 was considered to be a statistically significant difference.

## Authors' contributions

TY conceived of the study, and participated in its design. SY directed RNA interference, PCR, immunoassays. MY directed ALP staining and activity, and Mineralization assays. MY conceived of the study, and participated in its design. TS conceived of the study, and participated in its design and coordination. All authors read and approved the journal manuscript.
